# A Butyrate-Yielding Dietary Supplement Prevents Acute Alcoholic Liver Injury by Modulating Nrf2-Mediated Hepatic Oxidative Stress and Gut Microbiota

**DOI:** 10.3390/ijms25179420

**Published:** 2024-08-30

**Authors:** Qi Xu, Mei Guo, Haidi Wang, Haitao Liu, Yunbo Wei, Xiao Wang, Charles R. Mackay, Quanbo Wang

**Affiliations:** 1 Key Laboratory for Natural Active Pharmaceutical Constituents Research in Universities of Shandong Province, School of Pharmaceutical Sciences, Shandong Analysis and Test Center, Qilu University of Technology (Shandong Academy of Sciences), Jinan 250353, China; xuqi8270@sina.com (Q.X.); mguo@qlu.edu.cn (M.G.); 15689476503@163.com (H.W.); 19863823759@163.com (H.L.); weiyunbo0211@126.com (Y.W.); wangx@sdas.org (X.W.); c.mackay@me.com (C.R.M.); 2 Department of Microbiology, Infection and Immunity Program, Biomedicine Discovery Institute, Monash University, Melbourne 3800, Australia

**Keywords:** butyrate-yielding diet, short-chain fatty acid, alcoholic liver injury, oxidative stress, gut microbiota

## Abstract

Alcoholic liver disease (ALD) is a globally prevalent form of liver disease for which there is no effective treatment. Recent studies have found that a significant decrease in butyrate was closely associated with ALD development. Given the low compliance and delivery efficiency associated with oral-route butyrate administration, a highly effective butyrate-yielding dietary supplement, butyrylated high-amylose maize starch (HAMSB), is a good alternative approach. Here, we synthesized HAMSB, evaluated the effect of HAMSB on acute ALD in mice, compared its effect with that of oral administration of butyrate, and further studied the potential mechanism of action. The results showed HAMSB alleviated acute ALD in mice, as evidenced by the inhibition of hepatic-function impairment and the improvement in liver steatosis and lipid metabolism; in these respects, HAMSB supplementation was superior to oral sodium butyrate administration. These improvements can be attributed to the reduction of oxidative stress though the regulation of Nrf2-mediated antioxidant signaling in the liver and the improvement in the composition and function of microbiota in the intestine. In conclusion, HAMSB is a safe and effective dietary supplement for preventing acute ALD that could be useful as a disease-modifying functional food or candidate medicine.

## 1. Introduction

Alcoholic liver disease (ALD) one of the most common forms of liver injury, covers a broad spectrum of disease states that range from alcoholic fatty liver, steatohepatitis and alcoholic cirrhosis to liver cancer [1]. ALD usually develops following excessive and acute intake of alcohol. In recent years, with the prevalence of alcohol consumption and the difficulty of abstinence, ALD has been becoming a rapidly spreading severe health problem worldwide [2]. Alcoholic cirrhosis accounts for more than 50% of cirrhosis-related mortality [3]. Given the lack of approved therapies for ALD, it is necessary to explore effective strategies to alleviate ALD, especially acute ALD induced by binge-drinking, in order to prevent the progression of ALD in an early stage.

The gut microbiota play a vital role in maintaining the health of the host. Short-chain fatty acids (SCFAs), including acetate, propionate and butyrate, are derived from the fermentation of non-digestible carbohydrates by gut microbiota, which is a major link between microbiota and host physiology. Currently, the anti-inflammatory and health-promoting homeostasis-related activities of SCFAs in the intestines have attracted increasing attention. Accumulating evidence has shown that SCFAs are beneficial for the prevention or treatment for many diseases (at least in animal models), such as colitis, type 1 diabetes, arthritis, hypertension and Alzheimer’s disease [4,5,6,7,8]. Of note, recent studies supported the hypothesis that altered composition of the intestinal microbiota, particularly the decrease in SCFAs-producing bacteria, is closely associated with ALD progression [9]. Among these SCFAs-producing bacteria, butyrate-producing bacteria stand out as a result of their significant decrease in abundance [10]. In line with this, a similar significant decrease in fecal butyrate was observed in patients with ALD [11]. It is reported that oral administration of butyrate or tributyrin (a prodrug of butyrate) could ameliorate chronic ALD in mice [12,13]. However, it is still unclear whether butyrate shows a preventive effect against acute ALD. In addition, due to poor compliance and the low efficiency of butyrate delivery targeting the colon [13,14], oral administration of butyrate or tributyrin is not an ideal therapeutic strategy for ALD.

Butyrylated high-amylose maize starch (HAMSB), assembled from butyrate groups and high-amylose maize starch (HAMS) through ester bonds, is regarded as a stable and highly efficient mechanism for colon-targeted delivery of butyrate because HAMS is a resistant dietary fiber that can be broken down only by gut microorganisms in the colon. As such, HAMSB can combine the advantages of butyrate and prebiotics. It has been shown that the cecal and distal colonic butyrate concentrations were remarkably increased in mice fed with HAMSB [15], contributing to the restoration and maintenance of gut health. Furthermore, HAMSB has been used to prevent metabolic and immune-mediated diseases [5,16]. However, the protective effect of HAMSB with regard to acute ALD and whether this effect is better than that of oral butyrate remains unknown.

Here, we synthesized HAMSB and designed a specific diet based on HAMSB to model the sustained absorption of butyrate from the colon through bacterial fermentation of dietary fiber. Next, we evaluated the protective effect of HAMSB in acute ALD mice, then verified its superior effect in improving ALD compared to HAMS and butyrate and further explored the underlying mechanism in two important locations, the liver and the intestine.

## 2. Results

### 2.1. Characterization and SCFAs Delivery Effect of HAMSB

**Figure 1:**
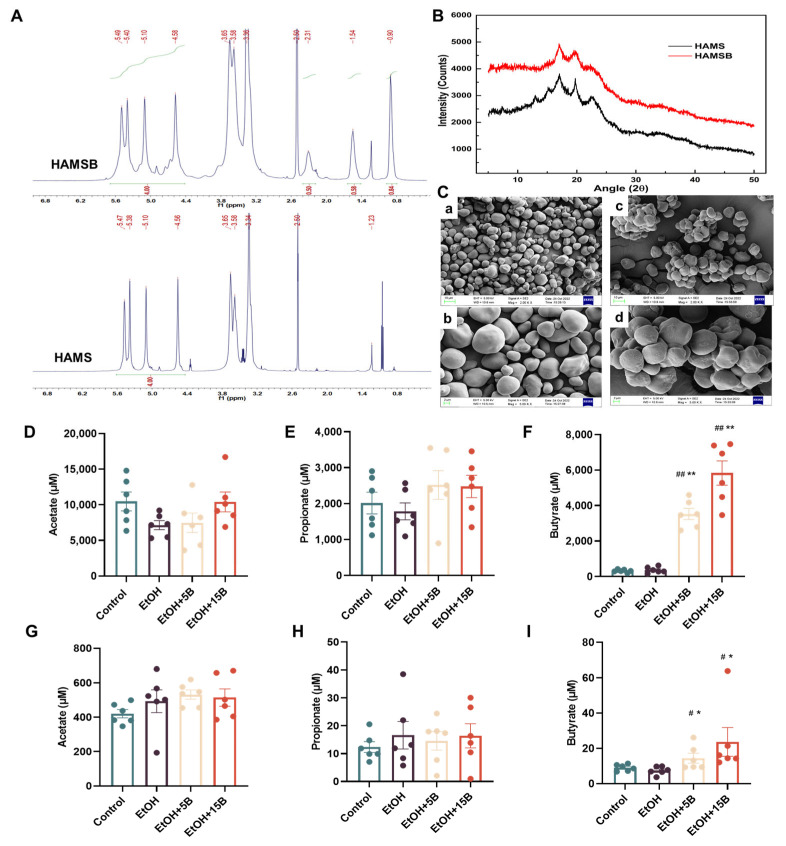
The characterization of HAMSB and SCFAs delivery by HAMSB. (**A**) 1H NMR spectra of native starch (HAMS) and HAMSB with a DS of 0.28. (**B**) X-ray diffraction patterns of HAMS and HAMSB. (**C**) SEM of starch samples with different magnifications: (a) HAMS, ×2000, (b) HAMS, ×5000, (c) HAMSB, ×2000 and (d) HAMSB, ×5000. (**D**–**F**) Concentrations of acetate, propionate and butyrate in feces of acute ALD mice. (**G**–**I**) Concentrations of acetate, propionate and butyrate in serum from the portal venous blood of acute ALD mice. Data are presented as mean ± SEM. (#) *p* < 0.05 and (##) *p* < 0.01 versus Control group; (*) *p* < 0.05 and (**) *p* < 0.01 versus EtOH group.

First, the physicochemical characteristics of HAMSB were assessed. As presented in Figure 1A, compared to the ^1^H NMR spectrum of native starch (HAMS), the spectrum of HAMSB showed the occurrence of the new peaks at 0.9 ppm (methyl), suggesting that an acylation of HAMS with a DS of 0.28 had been achieved. The crystalline structure of HAMSB was investigated by XRD (Figure 1B), the result showed that there was no significant difference in the peak position between HAMSB and HAMS, which indicated that the crystalline structure was not destroyed with the butyrylation. In addition, the morphological characteristics of HAMS and HAMSB are presented in Figure 1C. The imaging HAMS showed round, single granules with smooth surfaces, while that of HAMSB showed clusters of granules with less-smooth surfaces. This difference in morphology can be attributed to the starch gelatinization induced by acylation. Next, the butyrate-delivery efficiency of HAMSB was explored by GC-MS to determine its possible effect sites in acute ALD mice. As shown in Figure 1D–F, although the fecal acetate and propionate levels did not show significant differences among different groups, the fecal butyrate level in the HAMSB-treatment groups was elevated dramatically compared with that in the HAMS-treatment groups. Moreover, an increased butyrate level was observed with the increase in the content of HAMSB added in the diet, confirming the dose-dependence of butyrate release by HAMSB. Consistently, the concentration of butyrate, rather than those of acetate and propionate, in portal venous blood was also maintained at a higher level in HAMSB-supplemented groups than in other groups (Figure 1G–I). These findings implied that HAMSB played its role in mice by acting on the liver and intestines.

### 2.2. Effect of HAMSB on Liver Function in Acute ALD Mice

**Figure 2:**
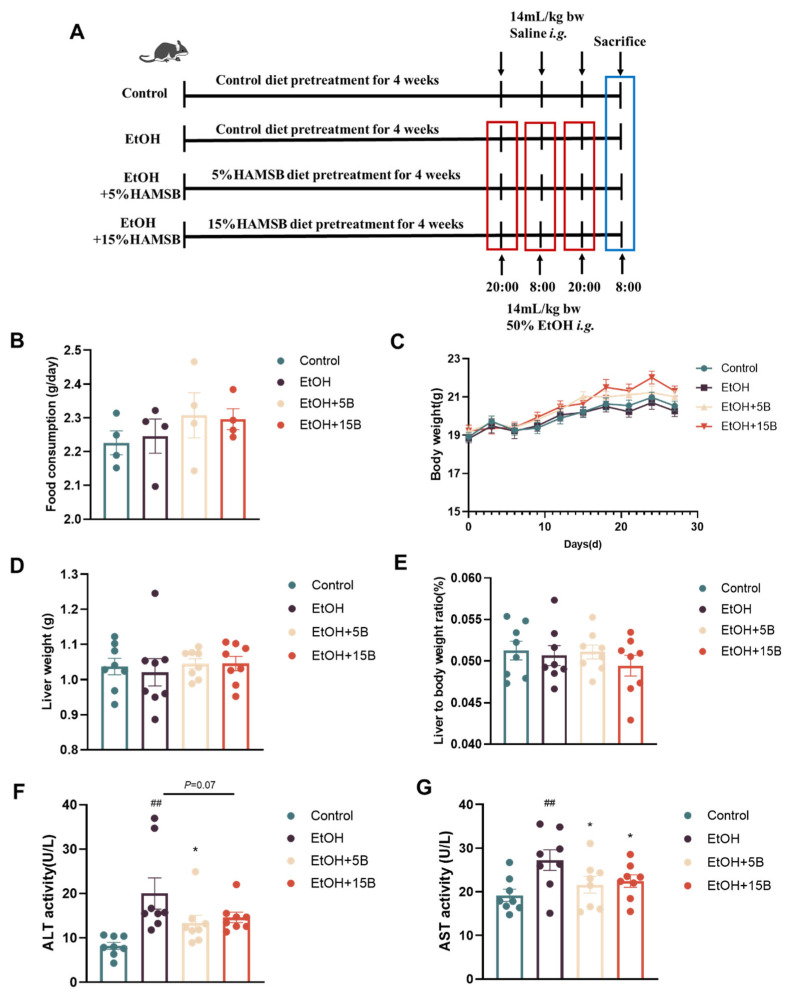
Effects of HAMSB on liver function in acute ALD mice. (**A**) Experimental design. (**B**) Food consumption by the mice. (**C**) Changes in body weight. (**D**) Liver weights of mice. (**E**) Liver-to-body-weight ratio. (**F**) The activity of ALT. (**G**) The activity of AST. Data are presented as mean ± SEM. (##) *p* < 0.01 versus Control group; (*) *p* < 0.05 versus EtOH group.

The timeline of diet intervention in the mice with acute ALD is shown in Figure 2A. Given that the basic profiles of mice can be used to evaluate the safety of HAMSB in vivo, the food consumption and body weight of the mice were recorded during the feeding period. As depicted in Figure 2B,C, HAMSB supplementation was not toxic to mice, as is evidenced by the absence of significant differences in food consumption and body weight among four groups. Subsequently, reliable indicators, including liver index and serum ALT and AST activities, were examined to measure the liver function. The results showed that a single binge of alcohol remarkably increased serum ALT and AST activities but did not influence the liver weight or liver index of mice (Figure 2D,E). After supplementation with different concentration of HAMSB, the levels of ALT and AST in mice were dramatically reduced (Figure 2F,G), indicating that HAMSB could alleviate the ethanol-induced impairment of liver function.

### 2.3. Effect of HAMSB on Liver Steatosis in Acute ALD Mice

**Figure 3:**
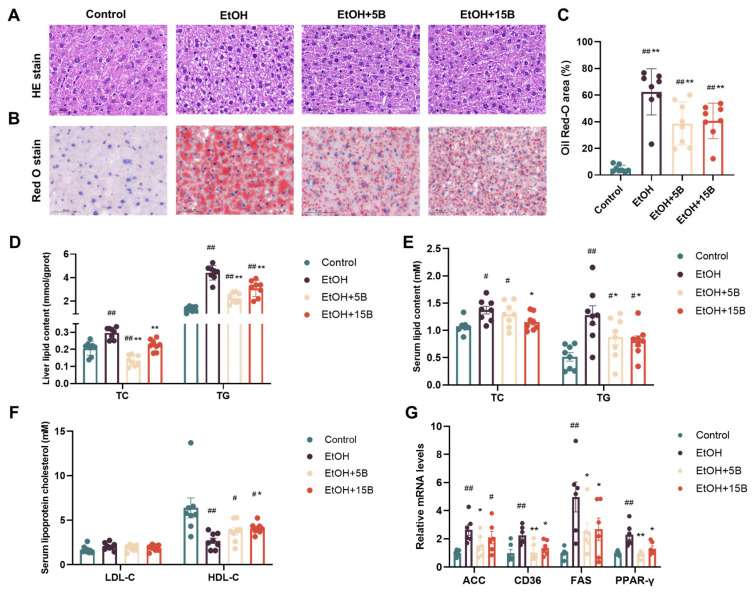
Effect of HAMSB on hepatic histological alterations and lipid content. (**A**) HE staining of liver-tissue slices (scale bar, 50 μm). (**B**) Oil Red O staining of liver-tissue slices (scale bar, 50 μm). (**C**) Quantification of the Oil Red-O-positive area. (**D**) Hepatic lipid levels, including TC and TG. (**E**) Serum lipid levels, including TC and TG. (**F**) Serum lipoprotein cholesterol levels, including LDL-C and HDL-C. (**G**) The mRNA expression levels of ACC, CD36, FAS and PPAR-γ in the liver were determined by qRT-PCR with normalization to GAPDH. Data are presented as mean ± SEM. (#) *p* < 0.05 and (##) *p* < 0.01 versus Control group; (*) *p* < 0.05 and (**) *p* < 0.01 versus EtOH group.

The fat accumulation in the liver was examined by histological and biochemical analysis to investigate the effect of HAMSB on liver steatosis. As shown by HE (Figure 3A) and Oil Red O staining (Figure 3B,C), in comparison to the control group, the obvious macrovesicular steatosis, which manifested in the accumulation of large lipid vacuoles and lipid droplets in the liver, were observed in the EtOH group. However, HAMSB pretreatment could significantly ameliorate these pathological changes, with only slight microvesicular steatosis present in the livers of the mice. In line with the results of the histological analysis, the increase in liver lipid content (TG and TC) induced by alcohol consumption was gradually returned to normal levels via the administration of HAMSB (Figure 3D). Additionally, serum TC, TG and HDL-C levels in the EtOH group were also normalized by HAMSB supplementation. There was no significant difference in the level of serum LDL-C between the alcohol and HAMSB treatment groups (Figure 3E,F). Moreover, HAMSB supplementation inhibited the upregulated expression of lipid-synthesis-related genes (ACC, FAS) and lipid-transport-related genes (CD36, PPARγ) caused by alcohol (Figure 3G). Notably, in these results, the 15% HAMSB did not perform better than the 5% HAMSB in preventing alcohol-indued liver steatosis.

### 2.4. Comparison of the Effects of HAMSB and Butyrate in Alleviating ALD

**Figure 4:**
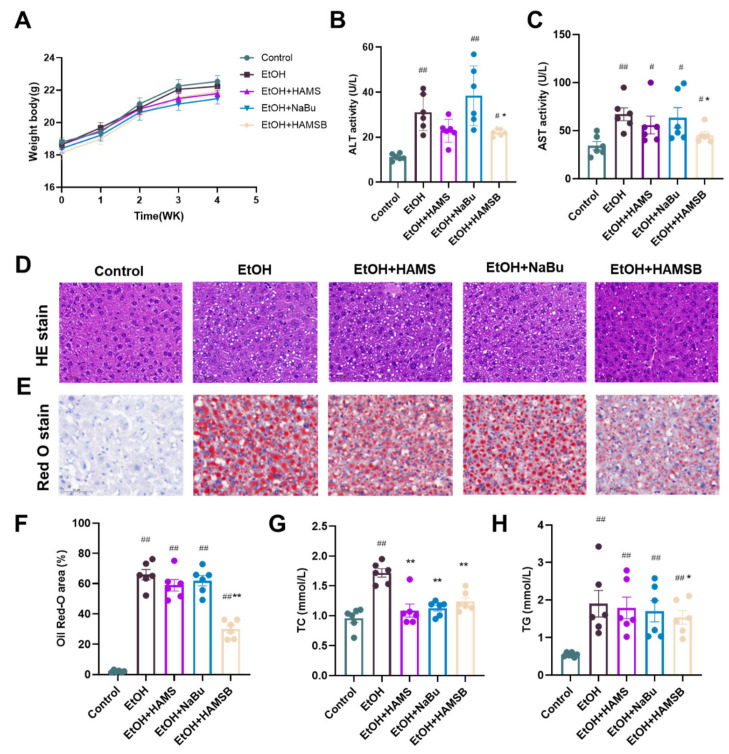
Comparison of the effects of HAMSB and butyrate in alleviating ALD. (**A**) The body weights of mice with different treatments. (**B**,**C**) The levels of ALT and AST in serum. (**D**,**E**) Representative images of histological liver sections after (**D**) HE staining and (**E**) Oil Red O staining (scale bars, 50 μm). (**F**) Quantification of the Oil Red O-positive area. (**G**,**H**) The contents of TC and TG in serum. Data are presented as mean ± SEM. (#) *p* < 0.05 and (##) *p* < 0.01 versus Control group; (*) *p* < 0.05 and (**) *p* < 0.01 versus EtOH group.

To further verify the superior effect of HAMSB in improving ALD, liver function and steatosis in ALD mice treated with HAMS, sodium butyrate (NaBu, with consistent butyrate administration of HAMSB) and HAMSB were compared; the results are summarized in Figure 4. Among different groups, the body weights of the mice were basically the same, revealing that different treatments had no significant influence on mouse body weight (Figure 4A). However, after ethanol exposure, mice treated with HAMSB displayed more significant improvement in liver function compared with mice in HAMS and NaBu treatment groups, as evidenced by the more significant decreases in ALT and AST levels in those mice (Figure 4B,C). As to liver steatosis, histologic analysis showed the accumulation of lipid vacuoles and lipid droplets in the HAMSB treatment group was less than that in other groups (Figure 4D–F). Although the serum TC content was reduced remarkably in ALD mice treated with HAMS, HAMSB and NaBu, only the HAMSB-fed group had a serum TG content lower than that in the ethanol-fed group (Figure 4G,H). These results strongly suggested that the administration of HAMSB improved ALD symptoms without relying on HAMS, and its effect was better than that of oral administration of butyrate in a form such as NaBu.

### 2.5. Effect of HAMSB on Oxidative Stress in the Liver of Acute ALD Mice

**Figure 5:**
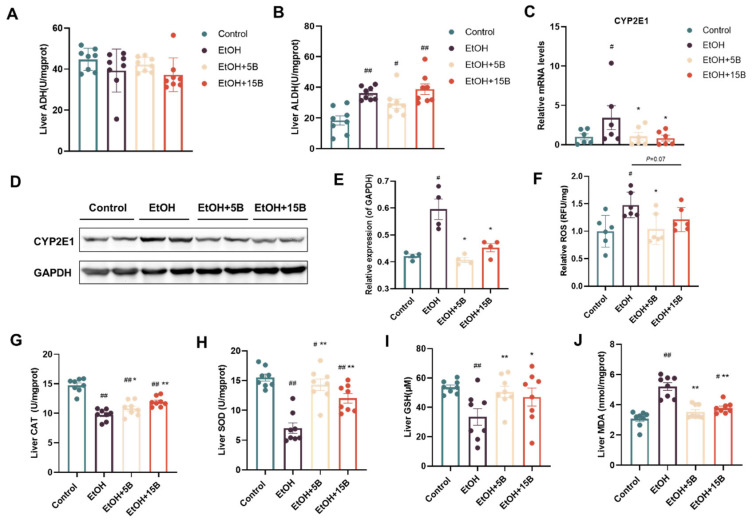
Effect of HAMSB on oxidative stress in the liver in acute ALD mice. (**A**,**B**) ADH and ALDH activities in the liver. (**C**) CYP2E1 gene expression in the liver. (**D**) Representative image of western blot analysis and (**E**) quantification of CYP2E1 protein expression in the liver, normalized to GAPDH. (**F**) ROS level in the liver. (**G**) CAT activity, (**H**) SOD activity, (**I**) GSH activity and (**J**) MDA level in the liver. Data are presented as mean ± SEM. (#) *p* < 0.05 and (##) *p* < 0.01 versus Control group; (*) *p* < 0.05 and (**) *p* < 0.01 versus EtOH group.

Generally, ethanol absorbed in the liver is converted to acetaldehyde by ADH or metabolized by CYP2E1 to produce ROS, thus resulting in liver damage [17]. Therefore, the effect of HAMSB on ethanol metabolization in the liver was evaluated. On the one hand, as displayed in Figure 5A,B, alcohol consumption enhanced ALDH activity but not ADH activity in the liver. Neither ALDH activity nor ADH activity was influenced by HAMSB supplementation. On the other hand, qRT-PCR and western blot analysis revealed that the expression of CYP2E1 protein was significantly increased after alcohol administration and that this effect was reversed by HAMSB treatment (Figure 5C–E). Based on the above finding, it can be concluded that HAMSB mainly regulated CYP2E1 expression to control ethanol metabolization in the liver. Hence, several parameters, such as ROS, CAT, SOD, MDA and GSH, were assayed to examine the effect of HAMSB on oxidative stress associated with alcohol metabolization by CYP2E1. As shown in Figure 5F–J, acute alcohol exposure markedly increased the ROS and MDA levels and notably decreased the activities of CAT, SOD and GSH in the livers of exposed mice compared to the control group, implying the occurrence of oxidative stress in the liver in their livers. Conversely, these parameters were restored to various extents in HAMSB-fed groups. In summary, HAMSB could ameliorate alcohol-induced oxidative stress in the liver in the liver in mice.

### 2.6. Effect of HAMSB on Nrf2-Mediated Antioxidant Signaling in the Livers of Acute ALD Mice

**Figure 6:**
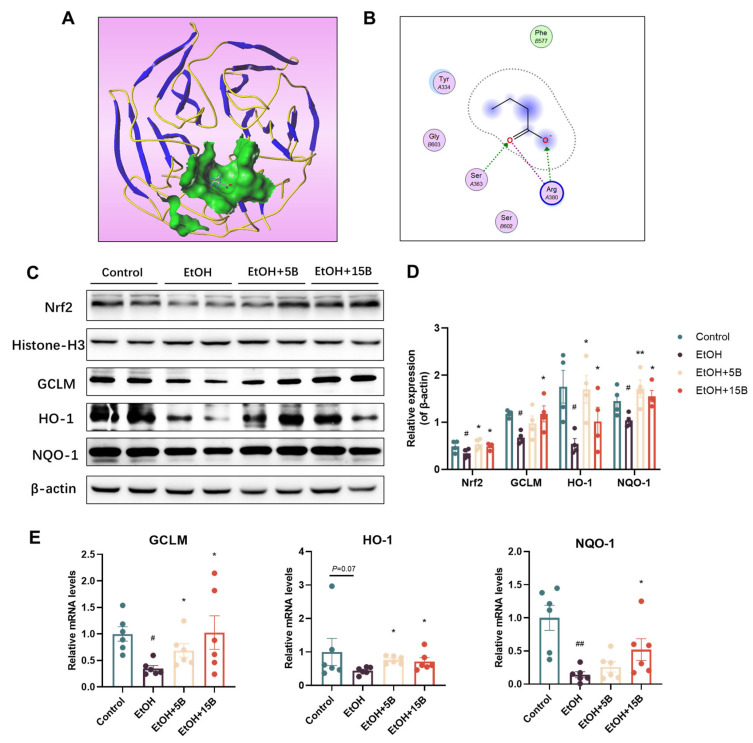
Effect of HAMSB on the Nrf2-mediated antioxidant signaling pathway. (**A**,**B**) Interaction of butyrate and Keap-1 at the active domain. The predicted 3D (**A**) and 2D (**B**) binding mode of butyrate with Keap-1. (**C**) Representative image of western blot analysis and (**D**) quantification of nuclear Nrf2, GCLM, HO-1 and NQO-1 protein expression in the liver. (**E**) The mRNA expression of GCLM, HO-1, NQO-1 in the liver. Values of these genes from qRT-PCR analysis are normalized to the expression of GAPDH. Data are presented as mean ± SEM. (#) *p* < 0.05 and (##) *p* < 0.01 versus Control group; (*) *p* < 0.05 and (**) *p* < 0.01 versus EtOH group.

To determine whether HAMSB alleviated oxidative stress by activating the Nrf2-midiated antioxidant pathway, we first analyzed the potential binding between butyrate and Keap-1, which is the endogenous negative regulator of Nrf2. The docking model showed that butyrate could fit well into the pockets of the Keap1 protein by generating hydrogen-bonding interactions, especially between the two oxygen atoms of the carboxyl group and Ser363 and Arg380 (Figure 6A,B). Next, we examined Nrf2 and its downstream proteins using western blot and qRT-PCR analyses. As shown in Figure 6C,D, Nrf2 protein expression was remarkably reduced in the nuclear fraction of alcohol-fed mouse liver compared to that of control mice. As expected, supplementation with HAMSB resulted in increased Nrf2 translocation into the nucleus as compared to the alcohol-fed group. Moreover, Nrf2 target proteins, including GLCM, HO-1 and NQO-1, were also significantly downregulated upon exposure to alcohol. After pretreatment with HAMSB, this reduction was abolished to some extent. Meanwhile, this result was further confirmed by qRT-PCR analysis. As shown in Figure 6E, alcohol-induced downregulation of the mRNA expression of GLCM, HO-1 and NQO-1 was improved by HAMSB treatment. These data provide evidence that HAMSB can competitively bind Keap1 and thus activate Nrf2 and its target genes, which are involved in the antioxidant defense system, which contributes to the amelioration of oxidative stress in the liver.

### 2.7. Effect of HAMSB on Diversity and Structure of Intestinal Microbiota in Acute ALD Mice

**Figure 7:**
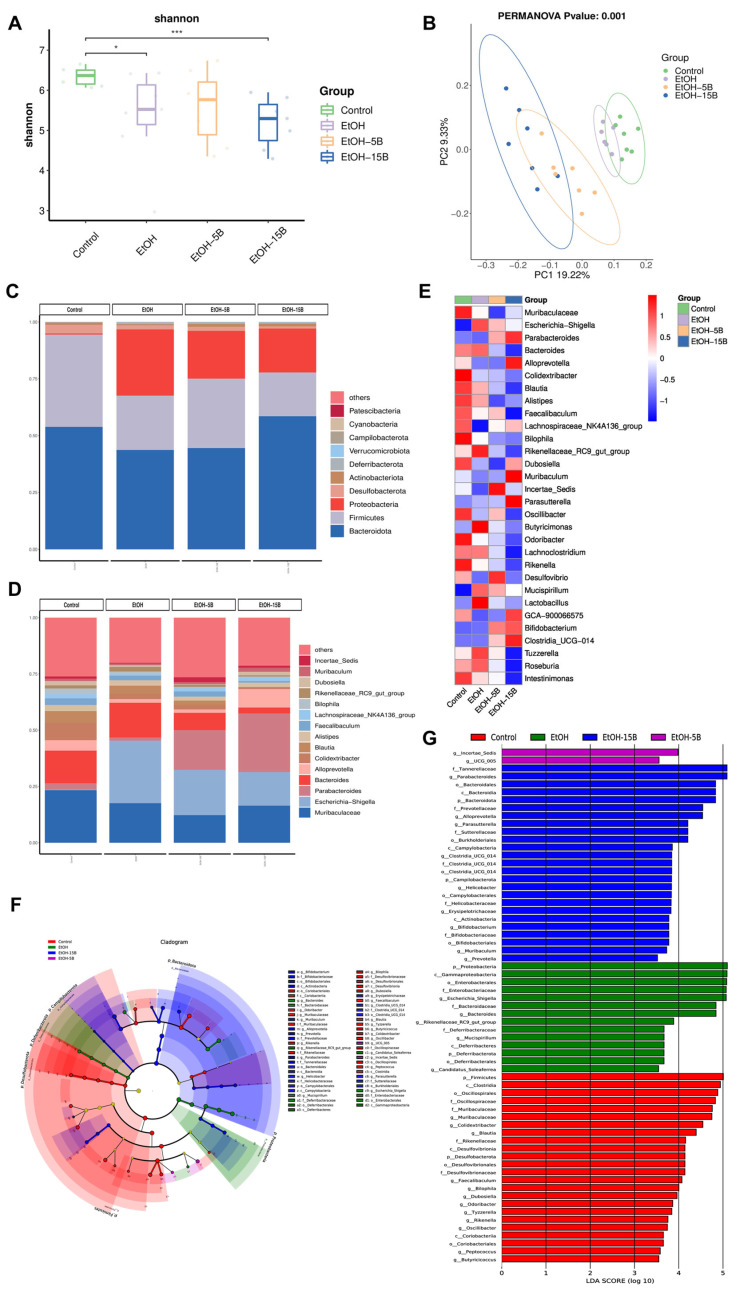
Effect of HAMSB on intestinal microbiota in acute ALD mice. (**A**) α-diversity index (Shannon index). (**B**) β-diversity was determined based on unweighted Unifrac principal coordinate analysis (PCoA). (**C**,**D**) The taxonomic profiles of the microbial communities at the phylum level (top 10 phyla) and genus-level (top 15 genera) in each group. (**E**) Heat map representing the intestinal microbial changes at the genus level in four groups. (**F**) Cladogram generated from the LEfSe. (**G**) Chart generated from the LEfSe. (*) *p* < 0.05, (***) *p* < 0.001.

Intestinal microbiota dysbiosis induced by alcohol consumption is associated with the progression of ALD. Therefore, changes in the intestinal microbiota were examined using 16S rDNA gene sequencing. As shown in the Venn diagram (Figure S1A), 242 ASVs of the total richness of 1302 ASVs were shared among all of the groups, while the unique ASVs in the Control, EtOH, EtOH + 5B and EtOH + 15B groups numbered, respectively, 201, 156, 200 and 191. The α-diversity analysis showed that the Shannon and Chao1 index values (Figure 7A and Figure S1B) in the EtOH group were lower than those in the control group, and it was further reduced after 15% HAMSB treatment. This result indicated that a high dose of HAMSB decreased the diversity of the gut microbiota. Meanwhile, the β-diversity of microbial communities was compared by PCA, PCoA and NMDS analyses. As shown in Figure 7B and Figure S1C,D, there were different clusters among the four groups, suggesting obvious alternations caused by HAMSB in the overall community of intestinal flora. Then, the influence of HAMSB on the structure of the intestinal microbiota at different taxonomic levels was further examined. At the phylum level (Figure 7C), it could be found that Bacteroidota, Firmicutes, Proteobacteria and Desulfobacterota dominated the intestinal microbiota of mice. Compared with the EtOH group, the HAMSB-supplemented group had significantly increased abundances of Bacteroidota, Actinobacteriota and Verrucomicrobiota but markedly decreased abundances of Proteobacteria and Desulfobacterota (Figure S1E). At the family level, more abundant Enterobacteriaceae and Lactobacillaceae and scarcer Lachnospiraceae and Ruminococcaceae were observed in the EtOH group than in the Control group, a difference that was reversed by HAMSB treatment (Figure S1F). At the genus level, as shown by the bar plot and heat map (Figure 7D,E), the relative abundances of *Escherichia-Shigella*, *Bacteroides* and *Blautia* were decreased, while those of *Parabacteroides*, *Bifidobacterium* and *Clostridia_UCG-014* were increased by HAMSB treatment. Interestingly, the relative abundance of *Lactobacillus* was higher in the alcohol-fed group than in the control group, whereas HAMSB supplementation restored it to normal levels in a dramatic effect. This finding was contrary to some previously reported results but consistent with those of our previous study [18]. Subsequently, the specific bacterial taxa that were predominant in four groups were identified by LDA Effect Size (LEfSe) analysis. As shown in Figure 7F,G, *Parabacteroides*, *Alloprevotella, Parasutterella*, *Helicobacter*, *Bifidobacterium*, *Muribaculum* and *Prevotella* were more abundant and naturally played key roles in the 15% HAMSB treatment group. To sum up, HAMSB might attenuate ALD via regulation of the intestinal microbiota.

### 2.8. Effect of HAMSB on Functional Profiles of Gut Microbiota in Acute ALD Mice

**Figure 8:**
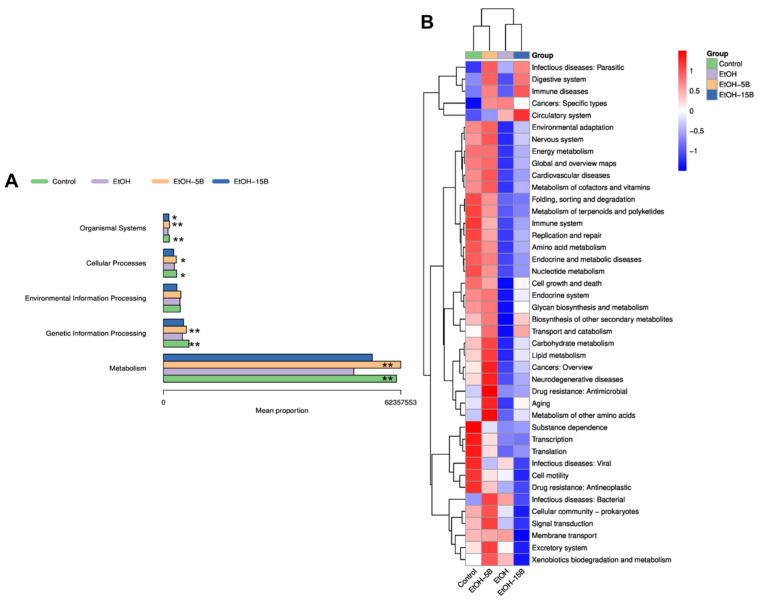
Effect of HAMSB on the functions of the intestinal microbiota in acute ALD mice. (**A**) Histogram of functional differences at level 1 of KEGG. (**B**) Heatmap of functional differences at level 2 of KEGG. * *p* < 0.05, ** *p* < 0.01 versus EtOH group.

To fully elucidate the regulating effect of HAMSB on intestinal microbiota, the functional potential of intestinal microbiota was predicted using PICRUSt2. As shown in Figure 8A, according to the functional predictions based on the KEGG database, gut microbes involved in metabolism, genetic-information processing, cellular processes and organismal systems were dramatically diminished by alcohol exposure. Specifically, the decreased function due to the alteration of the gut microbiota in the EtOH group could be presented in terms of environmental adaptation, energy metabolism, metabolism of cofactors and vitamins, metabolism of terpenoids and polyketides, amino acid metabolism, glycan biosynthesis and metabolism, biosynthesis of other secondary metabolites, carbohydrate metabolism and lipid metabolism (Figure 8B). However, these changes were restored by HAMSB treatment, especially by the 5% HAMSB treatment. Hence, it can be inferred that HAMSB also improved the function of the intestinal microbiota and thus alleviated acute ALD in mice.

## 3. Discussion

Alcoholic liver disease is considered to be a major cause of liver-related death worldwide, but, unfortunately, there are no effective therapies. The development of new agents capable of preventing or alleviating ALD is needed. Excessive alcohol consumption is the primary cause of ALD. The toxicity of alcohol can disrupt the proper functioning of important organs of the body, especially the liver and intestine, because most of the ingested alcohol is absorbed by the intestine and then enters the liver via the portal veinous circulation. In the present study, we synthesized HAMSB, a butyrate delivery system targeting the colon, which could enhance the level of butyrate not only in the colon, but also in the portal venous blood. HAMSB showed a superior preventative effect with regard to acute-alcohol-exposure-induced ALD in mice compared to oral administration of butyrate. Potential mechanisms are related to the reduction of oxidative stress in the liver and the modulation of microbiota dysbiosis in the intestine.

Liver is the main organ of alcohol metabolism; almost 80% of ingested alcohol in the body is metabolized by the liver [19]. In the liver, the damage to hepatocytes induced by acute alcohol consumption usually manifests in two pathophysiological responses. First, as transaminases are released into blood after hepatocyte injury, the activities of ALT and AST in serum are dramatically increased. ALT and AST are frequently used as an indicators of damage to liver function [20]. Additionally, alcohol intake destroys the homeostasis of lipid metabolism and leads to hepatic steatosis [21]. Our findings showed that HAMSB decreased serum ALT and AST activities, reduced hepatic lipid-droplet accumulation and normalized lipid metabolism in acute ALD mice. In conclusion, the pretreatment of HAMSB could ameliorate liver dysfunction and steatosis. Mechanistically, the alleviation of acute ALD in mice by HAMSB pretreatment relied on the reduction of oxidative stress. It is well-known that oxidative stress in the liver is the main mechanism implicated in all stages of ALD pathogenesis [22]. Generally, ingested alcohol is first oxidized to acetaldehyde by alcohol dehydrogenase (ADH). When alcohol drinking is excessive, the expression and activity of cytochrome P450 2E1 (CYP2E1), not ADH, are increased. Activated CYP2E1 promotes the conversion of alcohol to acetaldehyde, which is accompanied by the generation of a large amount of reactive oxygen species (ROS) [23]. The produced acetaldehyde is further oxidized to acetate by ALDH. The overproduction of ROS induces damage to cellular organelles and proteins, resulting in mitochondrial impairment, hepatic lipid accumulation and proinflammatory responses in the liver; these effects ultimately aggravate damage to hepatocytes [24,25]. Meanwhile, the antioxidant agents that hinder the accumulation of ROS in the liver, such as SOD and GSH, are inhibited after alcohol intake [26]. This imbalance between the overproduction of ROS and antioxidant defense capacity causes the oxidative stress. In this work, treatment with HAMSB inhibited the expression of CYP2E1 and notably decreased hepatic ROS and MDA levels, while CAT, SOD and GSH levels increased, thus lessening hepatic oxidative stress in acute ALD mice.

Furthermore, in the present study, we found that HAMSB inhibited hepatic oxidative stress via the regulation of Nrf2-mediated signaling. Nrf2 has long been identified as a positive regulator of intracellular adaptive antioxidant responses to oxidative stress and aids in the detoxification of a variety of toxins like acetaldehyde [27]. Nrf2 is highly expressed in the liver. Under normal conditions, Nrf2 is specifically bound by Keap1 to maintain its inactive status in the cytoplasm. Upon exposure to oxidative stress or other stimulations, Nrf2 is dissociated from Keap1 and thus translocated into the nucleus, where Nrf2 is dimerized with small musculoaponeurotic fibrosarcoma (sMAF) proteins to interact with antioxidant response elements (ARE) and finally activates the transcription of downstream target genes [28]. HO-1, NQO-1 and GCLM are the prototypical Nrf2 target genes and are involved in protection against oxidative stress [29]. Given the important role of Nrf2 in maintaining redox homeostasis, the relationship between Nrf2 and ALD has been investigated in many studies. A deficiency of Nrf2 in mice made them more susceptible to liver injury and mortality from binge alcohol exposure [30]. In contrast, the activation of Nrf2 by phytochemicals attenuated ALD in mice by enhancing antioxidant activity [31]. Therefore, NRf2-mediated antioxidant responses could be a viable target in the prevention of ALD pathogenesis [27]. Previous studies have revealed that interference in the interaction between Nrf2 and Keap1 caused the stabilization and the nuclear translocation of Nrf2 [32]. Hence, competitive binding with Keap1 protein is an alternative pathway to the activation of Nrf2. Our results showed that the butyrate released by HAMSB interacted with Keap1 and promoted the dissociation of Keap1 from Nrf2, which led to the nuclear translocation of Nrf2 and subsequently increased the expression of HO-1, NQO-1 and GCLM. This could explain why HAMSB inhibits oxidative stress in the liver.

The intestine is the first organ impacted by alcohol. In the intestine, alcohol is directly associated with compositional changes in the intestinal microbiota [33]. Numerous studies have revealed that alcohol induces the overgrowth of potentially pathogenetic taxa (such as Gram-negative bacteria) and depletion of some potentially beneficial autochthonous taxa (such as *Lachnospiraceae* and *Ruminococcaceae*) [34], as well as microbiome-derived metabolomics dysfunction [11]. Levels of SCFAs, bile acid and indole compounds were reduced during ALD progression. This alcoholism-based microbiota dysbiosis is closely associated with increased intestinal permeability, contributing to the translocation of harmful bacteria and microbial products from the intestine to the liver, where they pose a threat to hepatocytes [35]. In conclusion, homeostasis of the intestinal flora is important for the prevention and treatment of ALD [11,36]. Currently, limited studies have evaluated the role of HAMSB in protecting against gut microbiota dysbiosis in response to acute alcohol consumption. In this study, the correction of gut microbiota dysbiosis by HAMSB supplementation was reflected in the microbiota community structure and microbiota function. With regard to microbiota community structure, the increased numbers of Proteobacteria, *Enterobacteriaceae* and *Lactobacillaceae* caused by alcohol intake were reversed by HAMSB supplementation. This bacterial overgrowth can aggravate intestinal permeability and cause deterioration of liver function [37,38]. The Proteobacteria are highly studied gram-negative bacteria and have been recognized as a biomarker for gut dysbiosis [39]. Additionally, the relative abundance of beneficial autochthonous taxa, including *Ruminococcaceae*, *Alloprevotella* and *Bifidobacterium*, was increased after HAMSB treatment, a change associated with alleviation of ALD [11,40,41,42]. Intriguingly, our study found results conflicting with those of other studies in terms of the relative abundance of *Lactobacillus* [34]. This finding is consistent with that of our previous study and warrants further exploration. Of note, we found that, for some specific bacteria, the effect of 5% HAMSB is better than that of 15% HAMSB, a finding consistent with some of the functional results for HAMSB mentioned above. It is inferred that optimal supplementation with butyrate should be limited within a certain range. In terms of microbiota function, our data suggest that HAMSB supplementation improves the function of gut microbiota involved in energy, carbohydrate and lipid metabolism. A recent review highlighted the composition and function of gut microbiota, such as bile acid metabolism, as an important factor in alcoholic liver disease progression [33]. This is why HAMSB showed good potential for treating ALD.

## 4. Materials and Methods

### 4.1. Reagents and Materials

HAMS was purchased from Ingredion (Bridgewater, NJ, USA). Butyric anhydride was obtained from Bide Pharmatech Ltd. (Shanghai, China). Commercial assay kits for aspartate aminotransferase (AST), alanine aminotransferase (ALT), triglyceride (TG), cholesterol (TC), alcohol dehydrogenase (ADH), aldehyde dehydrogenase (ALDH), superoxide dismutase (SOD), catalase (CAT), malondialdehyde (MDA), reactive oxygen species (ROS), low-density lipoprotein cholesterol (LDL-C) and high-density lipoprotein cholesterol (HDL-C) were purchased from Nanjing Jiancheng Bioengineering Research Institute Co., Ltd. (Nanjing, China). A Reduced Glutathione (GSH) Content Assay Kit, Radioimmunoprecipitation Assay (RIPA) lysis buffer and a BCA Protein Assay Kit were purchased from Beijing Solarbio Science & Technology Co., Ltd. (Beijing, China). Primary antibodies including GCLM, HO-1, NQO-1, Histone-H3, GAPDH and all secondary antibodies were obtained from Affinity Biosciences (Cincinnati, OH, USA). CYP2E1 antibody and Nrf2 antibody were purchased from Abcam (Cambridge, MA, USA) and Cell Signaling Technology (Danvers, MA, USA), respectively.

### 4.2. HAMSB Preparation and Characterization

#### 4.2.1. Preparation of HAMSB

Butyrylated high-amylose maize starch was synthesized following previously established methods [43] with minor modifications. The 50% *w*/*w* starch suspension was obtained by mixing HAMS and distilled water, and the pH was adjusted to 8.0–9.0 by adding NaOH solution (2M). After that, butyric anhydride (32% based on the dry weight of starch) was added slowly. During the reaction, the pH of solution remained at 8.5. The whole reaction was conducted at room temperature. In the end, 0.5% HCl solution was used to adjust the pH of the starch slurry to 6.5. The mixture was washed, dried in an oven at 40 °C and finally ground though a 100-mesh sieve for future use.

#### 4.2.2. ^1^H Nuclear Magnetic Resonance (NMR) Spectroscopy

The ^1^H NMR spectra of HAMSB were measured according to the methods used in our previous study [44].

#### 4.2.3. Scanning Electron Microscopy (SEM) Observation

The starch samples were adhered to the sample stage and coated with a thin film of gold (10 nm) under vacuum conditions. The morphology of the starch was observed using a SEM (SUPRA™ 55, Zeiss, Hebron, KY, USA) at 10 kV acceleration voltage. Representative images with different magnifications were acquired.

#### 4.2.4. X-ray Diffraction (XRD) Measurement

The XRD analysis of the starch samples was performed using an XRD-EMPYREAN diffractometer (PANalytical B.V., Almelo, The Netherlands) at a scan rate of 4°/min within the 2θ range of 3–50°.

### 4.3. Animals and Treatments

C57BL/6J mice (female, 8 weeks old, 18–20 g) were obtained from Beijing River Laboratory Animal Technology Co., Ltd. (Beijing, China) and housed in cages in a controlled environment with 50 ± 10% relative humidity, 25 ± 2 °C temperature and 12 h light/12 h dark cycle throughout the study. After one week of acclimation, the mice were randomly divided into four groups: (1) control group (Control, *n* = 8), (2) acute alcoholic liver injury model group (EtOH, *n* = 8), (3) 5% HAMSB treatment group (EtOH + 5B, *n* = 8) and (4) 15% HAMSB treatment group (EtOH + 15B, *n* = 8). Mice were fed with the AIN-93G diet (Control group, EtOH group) or a modified AIN-93G diet with 5% or 15% corn starch replaced by HAMSB (EtOH + 5% HAMSB group, EtOH + 15% HAMSB group). The diet ingredients are shown in Table 1. After four weeks of feeding, mice were gavaged with 50% (*v*/*v*) ethanol (14 mL/kg body weight) to induce acute alcoholic liver injury, while the mice in the control group were gavaged with the same volume of saline. Then, the mice were fasted for 12 h and sacrificed for sample collection. Whole blood was centrifuged at 2500 rpm for 15 min to collect serum. Whole liver tissue was weighed to calculate the liver-to-body-weight ratio and then frozen or fixed for follow-up analysis. The feces were collected for 16s rDNA analysis. Next, in order to compare the effects of HAMSB, HAMS and butyrate in improving ALD, the mice were randomly divided into five groups: (1) control group (Control, *n* = 6), (2) acute alcoholic liver injury model group (EtOH, *n* = 6), (3) 5% HAMS treatment group (EtOH + HAMS, *n* = 6), (4) sodium butyrate treatment group (EtOH + NaBu, *n* = 6, sodium butyrate in drinking water with concentration of 3.85 mg/mL, with consistent butyrate intake dose in molar weight as 5% HAMSB treatment group) and (5) 5% HAMSB treatment group (EtOH + HAMSB, *n* = 6). The process for the animal experiments was as described above. Throughout the study, the animals’ food consumption was recorded once a week. The Animal Care and Use Committee of Shandong Analysis and Test Center endorsed the experimental procedures used in this study.

### 4.4. Biochemical Analysis

Liver tissues were homogenized with PBS at a ratio of 1 (g) to 9 (mL) to prepare 10% tissue homogenate. The supernatants were collected by centrifuging the tissue homogenate (2500 rpm, 4 °C, 10 min) for hepatic biochemistry analysis. Then, the levels of AST, ALT, TC, TG, LDL-C and HDL-C in serum and the contents of TC, TG, ADH, ALDH, CAT, SOD, GSH, MDA and ROS in the liver tissues were determined according to the manufacturers’ instructions for the assay kits.

### 4.5. Histological Analysis

For hematoxylin−eosin (HE) staining, the liver tissues were fixed with 4% paraformaldehyde, embedded in paraffin and sectioned into 3 μm thick slices. Next, the slices were subjected to hematoxylin−eosin (HE) staining [10]. For Oil Red O staining, liver tissues were frozen at −80 °C, cut into 5 μm sections, and stained with Oil Red O (Sigma, St. Louis, MO, USA) [10]. Finally, all tissue slices were scanned with Panoramic SCAN (3DHISTECH Ltd., Budapest, Hungary) and observed with CaseViewer software 2.3.

### 4.6. Quantification of Short-Chain Fatty Acids (SCFAs)

The concentration of SCFAs in feces and portal blood was determined by gas chromatography-mass spectrometry (GC-MS), as previously described [18]. In brief, approximately 20 mg feces were homogenized in 800 μL ultra-pure water. After centrifugation (10,000 rpm at 4 °C for 10 min), 100 μL supernatants were spiked with 10 μL 2-ethyl butyric acid (Sigma-Aldrich, St. Louis, MO, USA) as an internal standard and then mixed with 44 μL PBS and 280 μL PFBBr at 60 °C for 1.5 h. Next, the mixture was vortexed with 200 μL n-hexane for 2 min and centrifuged at 10,000 rpm at 4 °C for 10 min. The upper organic layer was transferred to a sample vial and analyzed by gas chromatography−mass spectrometry (Agilent Technologies, Santa Clara, CA, USA). The quantification was performed using the corresponding external calibration curves.

### 4.7. Western Blot Analysis

Approximately 20 mg of liver tissues was homogenized with 350 μL RIPA lysis buffer supplemented with 1 mM phenylmethylsulfonyl fluoride (PMSF) and then lysed on ice for 15 min. The supernatants were obtained by centrifuging the lysates at 12,000 rpm for 15 min, and the protein concentration was measured using the BCA Protein Assay Kit. Subsequently, supernatants were boiled with 1 × loading buffer at 95 °C for 10 min to prepare the protein sample. A total of 10 μg protein was fractionated on 8–12% sodium dodecyl sulfate-polyacrylamide gel (SDS-PAGE) and transferred onto PVDF membranes, which were blocked with 5% non-fat milk and incubated overnight at 4 °C with various primary antibodies. After being washed with TBST, the membranes were incubated with HRP-conjugated secondary antibodies and then observed using G:BOX Chemi XX9 (Syngen, London, UK). Protein bands were finally quantified by Image J 1.8.0 (Bethesda, MD, USA) and normalized to GAPDH or β-actin.

### 4.8. Real-Time Quantitative PCR

Total mRNA from the liver tissues was extracted and reverse-transcribed using the SPARKeasy Improved Tissue/Cell RNA Kit and SPARKscript II RT Plus Kit (SparkJade, Jinan, China) with reference instructions. cDNA was subjected to qPCR analysis with the SYBR Green qPCR Mix (SparkJade, Jinan, China). The primer sequences in this study are shown in Table S1, and all gene expression was standardized to GAPDH.

### 4.9. Sequencing to Determine Gut Microbiota Diversity 

Fresh feces of mice in different groups were snap-frozen and stored at −80 °C after collection. Bacterial DNA was isolated using a DNeasy PowerSoil kit (Qiagen, Hilden, Germany). DNA concentration and integrity were measured on a NanoDrop 2000 spectrophotometer (Thermo Fisher Scientific, Waltham, MA, USA) and by agarose gel electrophoresis, respectively. The V3-V4 hypervariable regions of the bacterial 16S rRNA gene were amplificated using universal primer pairs (343F, -TACGGRAGGCAGCAG; 798R, AGGGTATCTAATCCT). The purified PCR products were sequenced on an Illumina NovaSeq6000. Raw sequencing data were in FASTQ format. After trimming, paired-end reads were filtered for low-quality sequences, denoised and merged and chimeric reads were detected and cut off using DADA2 with the default parameters of QIIME2. At last, the software output the representative reads and the ASV abundance table. All representative reads were annotated and BLAST searched against the Silva database Version 138 (or Unite) (16s/18s/ITS rDNA) using the q2-feature-classifier with the default parameters. The sequencing and analysis of the 16S rRNA gene amplicon were conducted by OE Biotech Co., Ltd. (Shanghai, China).

### 4.10. Molecular Docking

Molecular docking of butyrate with Keap1 was carried out with the AutoDock 4.2.6 program. The Keap1 protein and butyrate structures were prepared using AutoDock Tools 1.5.6, and the corresponding pdbqt files were generated. The structure with the lowest docking energy was identified in two stages: (1) 2000 steps of optimization with the steepest descent method; (2) 2000 steps of further optimization by the conjugate gradient method. The active site of Keap1 was wrapped in the docking box. The parameters were set as follows: the number of grid points in the XYZ of the grid box was set to 60 × 60 × 60, the grid spacing was 0.5 Å, the number of GA runs was set to 100 and the remaining parameters were set to their default values. The molecular-docking result was generated using PyMol (http://pymol.sourceforge.net/, accessed on 6 December 2023).

### 4.11. Statistical Analysis

All data were expressed as mean ± SEM. Statistical analysis in this study was performed using ANOVA (one-way analysis of variance) followed by LSD (Fisher’s least significant difference) test (SPSS 19.0). A value of *p* < 0.05 was regarded as significant. The figures were generated using GraphPad 8.0 (San Diego, CA, USA).

## 5. Conclusions

Taken together, our results demonstrate that HAMSB, a modified starch that can be used for colon-targeted delivery of butyrate, exerted considerable preventive effects against acute ALD in mice and that this result could be dependent on the enhancement of butyrate levels in the liver and intestine. The underlying mechanism included anti-oxidative stress effects exerted by the activation of Nrf2-mediated signaling in the liver and modulation of microbiota dysbiosis in the intestine. This study provides evidence to support the potential use of HAMSB as a safe and effective candidate for alleviating acute-alcohol-exposure-induced hepatic injury.

## Figures and Tables

**Table 1:** Mouse diet ingredients.

Ingredients (g)	Control Diet		5% HAMSB Diet		15% HAMSB Diet	
	g	kcal	g	kcal	g	kcal
Casein	200	800	200	800	200	800
L-cystine	3	12	3	12	3	12
Corn starch	444	1776	422.5	1690	379.5	1518
HAMSB	0	0	50	86	150	258
Sucrose	100	400	100	400	100	400
Maltodextrin	45.725	182.9	50	200	50	200
Cellulose	50	0	50	0	50	0
Sunflower seed oil	70	630	70	630	70	630
Mineral mix S10022G	0	12	0	12	0	12
Vitamin mix V10037	10	40	10	40	10	40
Choline bitartrate	2.5	0	2.5	0	2.5	0
Total	960.225	3840.9	993	3858	1050	3858
	gm%	kcal%	gm%	kcal%	gm%	kcal%
Protein	21.1	21.1	20.4	21	19.3	21
Carbohydrate	62.6	62.5	63.7	62.6	65.7	62.6
Fat	7.9	16.4	7	16.3	6.7	16.3

## Data Availability

The data presented in this study are available on request from the corresponding author. The data are not publicly available due to privacy. The studies did not involve humans.

## Supplementary Materials

The following supporting information can be downloaded at: https://www.mdpi.com/article/10.3390/ijms25179420/s1.
