# Supplementary material for: A Butyrate-Yielding Dietary Supplement Prevents Acute Alcoholic Liver Injury by Modulating Nrf2-Mediated Hepatic Oxidative Stress and Gut Microbiota
Source: Int J Mol Sci. 2024 Aug 30;25(17):9420. doi: 10.3390/ijms25179420 (PMC11395132; doi:10.3390/ijms25179420)
Supplement: Supplementary file 1 [file ijms-25-09420-s001.zip › ijms-3139681-supplementary.pdf]

Table S1. Sequence of Primer Used for qRT-PCR.

gene name	primer sequences（5’-3’）
ACC	F: 5’-ATGGGCGGAATGGTCTCTTTC-3’
	R: 5’- TGGGGACCTTGTCTTCATCAT-3’
CD36	F: 5’- TTGTACCTATACTGTGGCTAAATGAGA -3’
	R: 5’- CTTGTGTTTGAACATTTCTGCTT -3’
FAS	F: 5’-TATCAAGGAGGCCCATTTTGC-3’
	R: 5’-TGTTTCCACTTCTAAACCATGCT -3’
PPAR-γ	F: 5’- ATGTCTCACAATGCCATCAGGTT -3’
	R: 5’- GCTCGCAGATCAGCAGACTCT -3’
CYP2E1	F: 5’- CCAACTCTGGACTCCCTTTTAT-3’
	R: 5’-ACGCCTTGAAATAGTCACTGTA3’
HO-1	F: 5’- TGCAGGTGATGCTGACAGAGG -3’
	R: 5’- GGGATGAGCTAGTGCTGATCTGG -3’
GCLM	F: 5’- ACATTGAAGCCCAGGATTGG -3’
	R: 5’- CCCCTGCTCTTCACGATGAC -3’
NQO-1	F: 5’- TATCCTTCCGAGTCATCTCTAGCA -3’
	R: 5’- TCTGCAGCTTCCAGCTTCTTG -3’
GAPDH	F: 5’- AGGAGCGAGACCCCACTAACA -3’
	R: 5’- AGGGGGGCTAAGCAGTTGGT -3’

Figure S1

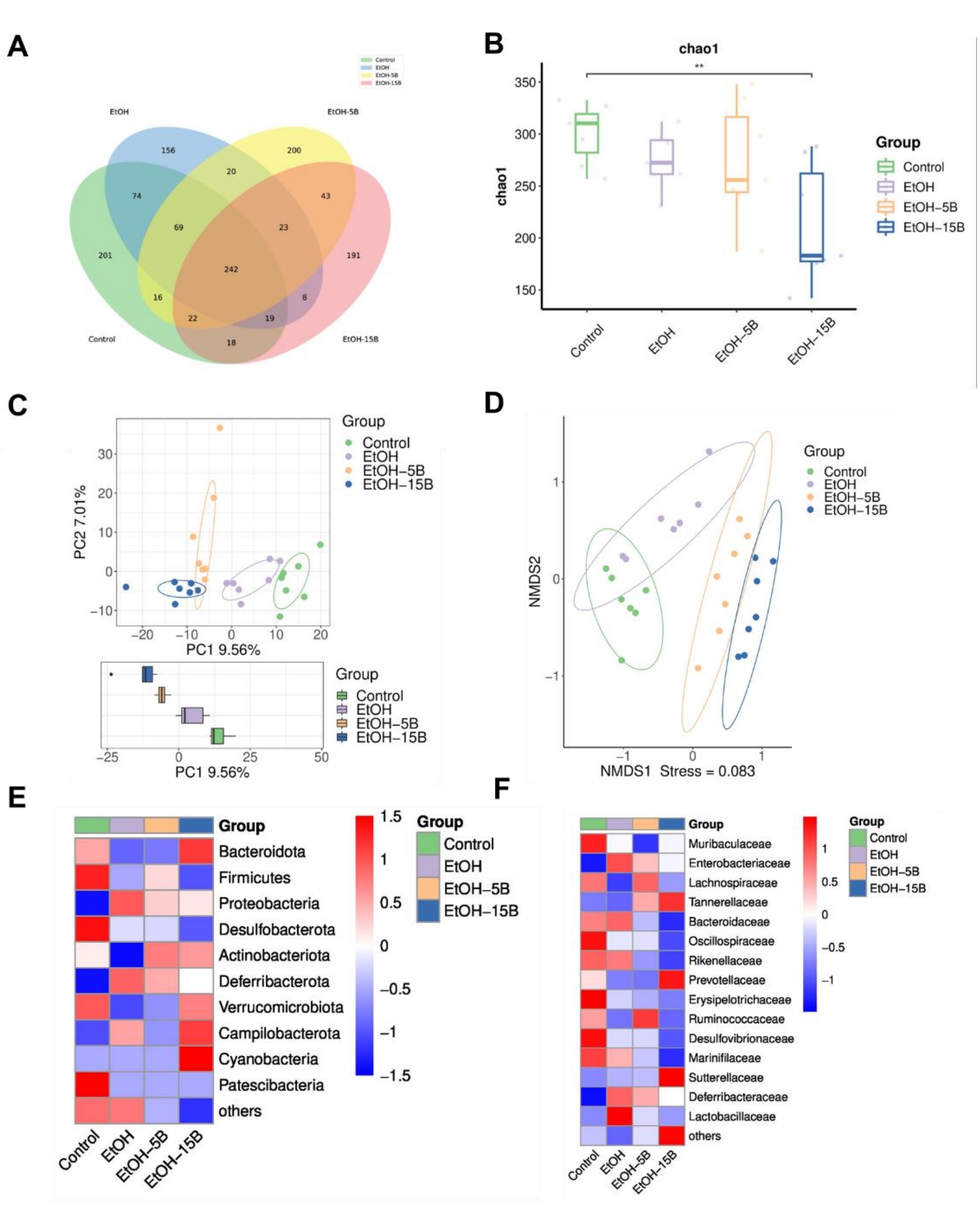

Figure S1. Effect of HAMSB on intestinal microbiota in acute ALD mice. (A) Venn diagram illustrated overlap of ASVs in intestinal microbiota. (B) α-diversity index (Chao 1 index). (C, D) β-diversity was determined based Principal Component Analysis (PCA) and using Bray Curtis distance algorithm based non-metric multidimensional scaling (NMDS). (E-F) Heat map representing the intestinal microbial changes at phylum level and family level in four groups.
